# Long-Term Regenerative Potential of Submandibular Glands in Albino Rats Following Radiotherapy: Role of Cytokeratin 17 Redistribution

**DOI:** 10.7759/cureus.81465

**Published:** 2025-03-30

**Authors:** Sherif S Hassan, Mashael Alqahtani

**Affiliations:** 1 Department of Oral Biology, Faculty of Dentistry, Al Azhar University, Cairo, EGY; 2 Department of Basic and Clinical Oral Sciences, College of Dental Medicine, Umm Al-Qura University, Makkah, SAU

**Keywords:** cytokeratin 17, intermediate filaments, radiotherapy, regeneration, submandibular gland

## Abstract

Background: Fractionated radiotherapy (RT) is a common approach for treating head and neck malignancies in which the salivary glands become included within the radiation field, causing xerostomia. This study aimed to evaluate the long-term effects of post-dose RT to determine the possibility of recovery of the submandibular gland (SMG) without intervention or further deterioration.

Methods: Twenty-seven albino rats were divided into three equal groups, each containing nine rats. The first group is the control group, which was not exposed to radiation. The second and third groups served as the experimental groups, receiving a total radiation dose of 30 Gy, divided into 5 Gy doses per day over six consecutive days. The SMGs were extracted and prepared for histopathological and immunohistochemical analysis, one month after irradiation for Group II and one year after irradiation for Group III.

Results: Immunohistochemical analysis of the control salivary gland revealed weak to mild staining for cytokeratin 17 (CK17) (intermediate filaments) in the cells of the duct system and serous acinar cells. The irradiated SMG exhibited significantly moderate to strong cytokeratin expressions in the duct cells (P=0.000 for Group II and P=0.025 for Group III) compared with the control. In contrast, the serous acinar cells in Group II exhibited significantly increased immunostaining for cellular keratin (P = 0.001) compared to the control group. Meanwhile, the acinar cells in Group III showed CK17 expression levels similar to those observed in the control group (P = 0.297).

Conclusion: CK17 expression differed significantly between the control and the second group, which underwent a one-month wait after the final radiation dose. In contrast, the third group, which underwent a one-year wait, showed intermediate results between the two groups, indicating a more advanced stage of recovery. Statistical analysis showed differing recovery dynamics, with ductal cells exhibiting slower recovery than acinar cells, which appeared more advanced in their healing. These findings highlight the progressive yet varied repair processes within the irradiated glands.

## Introduction

Radiotherapy (RT) is a common treatment modality for oral and para-oral malignancies, particularly squamous cell carcinoma, which accounts for 5% of all malignant tumors in the human body [1]. RT enhances clinical and aesthetic outcomes in patients with malignancies, whether used as the first treatment or after surgery for eliminating residual cancerous tissue [2]. Fractionated RT was the standard treatment for head and neck cancers, encompassing the salivary glands within the radiation field [3]. However, protecting the salivary gland tissues surrounding the radiation field is challenging, often resulting in significant damage that impairs their ability to secrete and flow saliva, leading to dry mouth [4].

The submandibular gland (SMG) is a major salivary gland that produces about 65% of the saliva in the oral cavity, serving protective and digestive functions and playing a vital role in oral and dental health [5,6]. Histologically, the SMG comprises parenchymal components, including ducts, serous and mucous acini, and myoepithelium, with richly vascularized connective tissue and unmyelinated nerve endings [5]. The acini appear as tubular structures formed of pyramidal secretory cells surrounding a central lumen, playing a crucial role in the production of primary saliva. The duct system is a branching network essential for modifying and transporting saliva from the acini to the larger ducts, ultimately draining into the oral cavity through the main duct, called Wharton's duct [7]. Also, SMG parenchyma contains a small population of stem cells linked to the duct system, sparking great interest in regenerative medicine for its potential to repair damaged glandular tissue [8]. Damage of SMG can be repaired through either the replication of viable acinar and ductal cells or the differentiation of stem cells [9]. Healing of damaged salivary glands, particularly after radiation injury, is dose-dependent and involves a complex coordinated process. This process includes various cellular and molecular mechanisms that work in tandem to restore glandular function and tissue integrity [10].

Saliva plays several important roles in the oral cavity, including moistening the mucous membranes, aiding speech, containing antimicrobial enzymes, and helping to maintain tooth integrity [11]. Xerostomia is one of the most harmful long-term side effects of multimodal treatment in patients with squamous cell carcinoma [12]. Xerostomia resulting from RT is primarily caused by the damaging effects on the parotid gland and SMG, which account for more than 80% of daily secreted saliva [5,13]. Xerostomia caused by RT inevitably leads to side effects on the oral and para-oral tissues. In the short term, these can result in issues such as dysphagia, loss of taste, and oral mucositis with speaking difficulties. On the other hand, long-term effects may develop over several months, impacting tooth structure and jaw bones and potentially delaying tooth eruption [12,14-17]. Additionally, many authors have observed that xerostomia induced by RT can develop after just a few treatment sessions and may persist for the remainder of the patient's life [18,19]. Significant histological changes of radiated SMG include a marked reduction in acinar size, acinar cell membrane damage, vacuolation, nuclear fragmentation, and cell necrosis [20].

The cytoplasm of the parenchymal cells contains a filamentous structure of three types: microfilaments (6µm thickness), intermediate filaments (cytokeratin in epithelial cells) of 15µm thickness, and microtubules (more than 25 µm) [5]. Animal cells possess six major intermediate filaments found throughout body tissues, with cytokeratin filaments specifically present in ectodermal acinar and ductal cells [21]. Intermediate filaments are essential for maintaining cellular structure, facilitating the movement of cell organelles and secretory vesicles within the cell, and enhancing cellular stability [22].

Immunohistochemistry (IHC) is a technique that expresses the cytokeratin filaments by forming the antigen-antibody complex, allowing the expression of the target molecules to be localized within their specific environments [23]. Immunoglobulin G (IgG) is a widely used antibody in IHC produced by immunizing an animal with a target antigen, which triggers a humoral response generating a monoclonal antibody. This antibody was then isolated from the animal to detect the expression of the antigen in human cells [24]. Cytokeratin 17 (CK17) immunoreactivity is an important biomarker due to its resistance to degradation, high antigenicity, stability, and consistent expression patterns in formalin-fixed, paraffin-embedded tissues. Several authors reported differences in immunoreactivity between irradiated and non-irradiated glands at doses ranging from 40 to 60 Gy, with stronger expression observed in a dose-dependent manner [10,25]. Our study was designed to evaluate the effects of a total RT dose of 30 Gy in a fractionated manner on the distribution of intermediate filaments in both ductal and acinar cells, comparing changes between one month and one year after the final dose to determine whether the outcomes lead to long-term gland destruction or recovery.

## Materials and methods

The study involved 27 healthy adult male Sprague Dawley albino rats, approximately five months old, with body weights ranging from 165 to 185 grams. The rats were provided with a balanced diet consisting of solid and soft foods with water available ad libitum. Before the experiment, mice were acclimatized in an animal care facility for at least one week and weighed daily to exclude any signs of illness or weight loss. The rats were housed in the laboratory animal facility and given therapeutic radiation doses at the Radiotherapy Department, Cancer Institute, Cairo University, Egypt. The rats were assigned to three equal groups of nine rats in each: Group I, which acted as a control and was subjected to general anesthesia with no radiation. Groups II and III received a total RT dose of 30 Gy using a linear accelerator external beam (Linacs), administered over six consecutive days at a rate of 5 Gy/day. Rats in Group II were sacrificed one month after the final radiation dose, while rats in Group III were allowed to survive for one year before being sacrificed.

Process of RT

**Figure 1:**
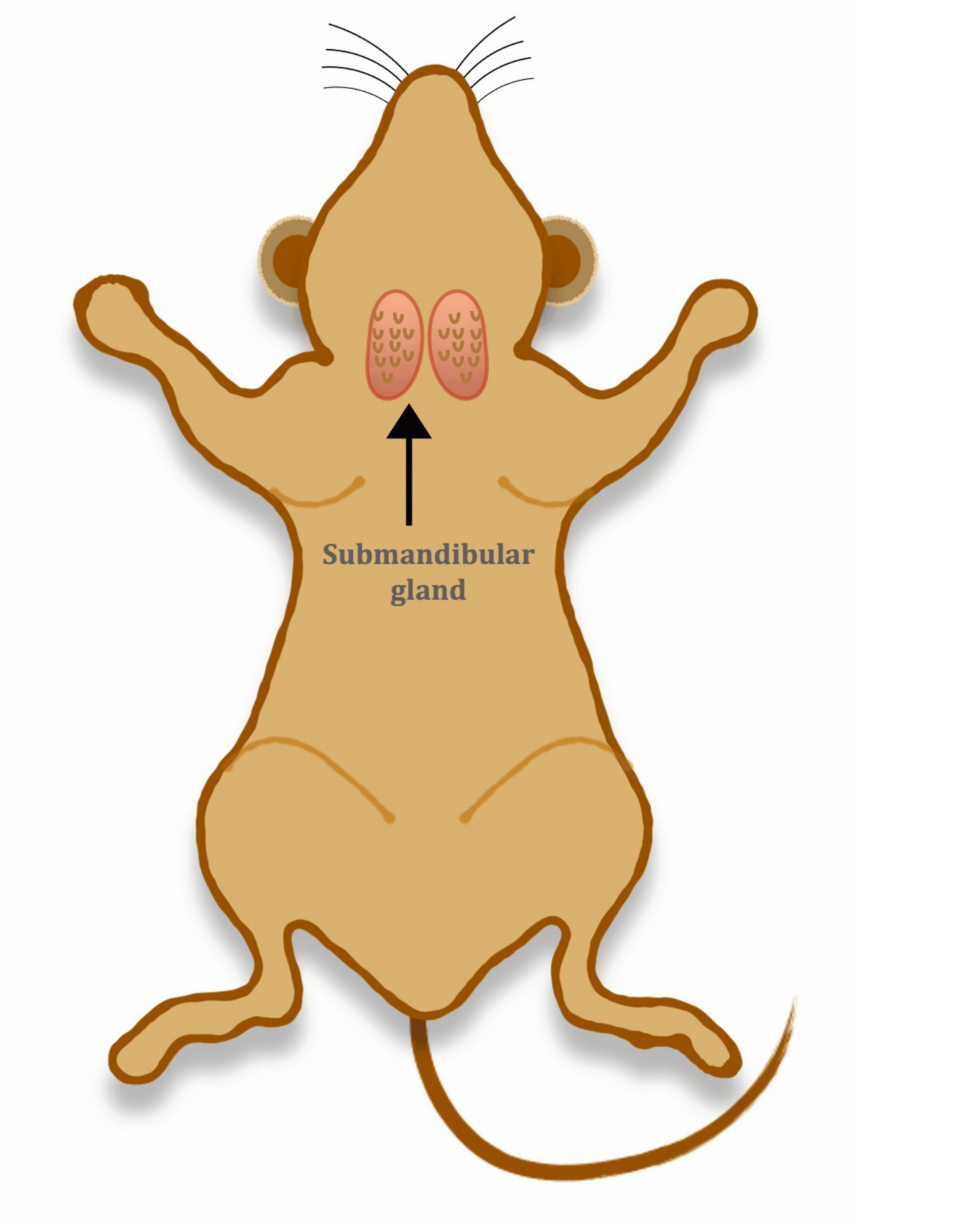
Anatomical location of right and left submandibular glands. This image is the author's own work.

On radiation days, all animals, including those in the control group, were placed under general anesthesia with sodium thiopental (30 mg/kg, AIPICO, Egypt). The control group was allowed to recover without radiation exposure. In the two irradiated groups, a 5 mm lead shield was positioned over the head and neck, leaving the targeted salivary gland area unshielded to protect other vital organs. A radiation dose of 5 Gy (1 Gy/min) was administered to a 3×3 cm field over the designated salivary gland area (Figure 1). Treatment was conducted from 8:00 a.m. to 2:00 p.m. for six consecutive days using an external-beam linear accelerator (Philips SL 75.5, Philips Medical Systems, Elekta, UK), a commonly used oral cancer treatment device. The accelerator operates at 235 kV and 15 mA and has a focal distance of 43 cm.

Process of histopathology

One month after the final radiation dose, the rats in the first and second groups were sacrificed via pentobarbital injection. The rats in the third group were sacrificed one year later using the same method. The submandibular salivary glands were carefully extracted and fixed in 10% neutral formalin for three days. Tissue staining was performed using paraffin-embedded tissue sections of 5-µm thickness. Effects were judged by hematoxylin and eosin (H&E) stained sections for histopathological examinations.

Immunohistochemical kits

The kits used were: anti-cytokeratin 17 monoclonal antibody (Host isotype: IgG) in liquid form; Universal Kit: Labelled streptavidin-biotin (LSAB)/horse radish peroxidase (HRP) kit with a content: a) Bottle (1) Hydrogen peroxide: 1x15 ml Hydrogen peroxide 3% solution in water; b) Bottle (2) Biotinylated link: 1x15 ml Biotin-labeled affinity in phosphate-buffered saline (PBS) containing carrier protein and 0.015 ml Sodium azide as a preservative; c) Bottle (3) Streptavidin-HRP: 1x15 ml Streptavidin conjugated to HRP in PBS containing carrier protein and antimicrobial agent; d) Bottle (4) Buffered substrate: 1x15 ml Imidazole-HCl (PH 7.5) containing hydrogen peroxide and antimicrobial agent; e) Bottle (5) Diaminobenzidine (DAB) Chromogen: 1x15 m DAB in chromogen solution; f) Accessories: Calibrated tube and Plastic Pasteur pipette; Antibody diluents: 15 ml Sodium azide, which was used as a diluent; Target retrieval solution: 500 ml of ready-to-use target retrieval solution to enhance the IHC staining. In tissue mounting: The tissue section must be mounted on a poly L-Lysine coated slide for staining procedures requiring target retrieval solution.

Process of IHC

IHC is a technique that detects intermediate filaments by forming antigen-antibody complexes, enabling the localization of target molecule expression within their specific environments. Paraffin sections 6 μm thick were mounted on coated microscopic slides, deparaffinized with xylene, rehydrated through a graded series of concentrated ethanol, and then incubated in methanol containing 0.3% hydrogen peroxide to inhibit endogenous peroxidase activity. Antigen retrieval was carried out by microwaving the slides for 5 minutes and incubating them in a block reagent for 10 minutes to reduce nonspecific staining. CK17 expression was detected using the monoclonal anti-CK17 E3 antibody (Sigma) through the LSAB method, with hematoxylin used as a counterstain. The positive expression of CK17 appeared as quantified spots with brown coloration in the ductal and acinar cells, and scores ranged from negative (0), weak (1), mild (2), moderate (3), and strong (4) [5,25].

Statistical analysis

Statistical analysis was performed to compare the three groups and assess whether there were significant differences in CK17 expression levels between the control and irradiated groups at various sacrifice time points. Data were written and calculated to be analyzed through IBM SPSS Statistics for Windows, Version 23 (Released 2015; IBM Corp., Armonk, New York, United States). The analysis aimed to determine if these differences reflected a trend of increasing gland destruction or a progression toward healing. Initially, we computed the statistical summary for each group, including the mean, median, standard deviation, and range, and then created a histogram to visualize the randomized data distribution. A one-way analysis of variance (ANOVA) was conducted to compare the results across the groups. A 95% confidence interval was applied, with a 5% margin of error, and statistical significance was defined as a p-value of ≤ 0.05. To assess significant differences in mean values between pairs of groups, we applied Tukey's honestly significant difference (HSD) and Dunnett's T3 tests.

## Results

Histopathological evaluation

Histological examination of the SMG in the control group showed lobular parenchymal tissue predominantly composed of closely packed serous, mucous, and mixed acini. These acini consist of pyramidal-shaped cells separated from the connective tissue stroma by a basement membrane. The duct system includes intercalated, striated, and excretory ducts, each displaying its normal characteristic features. Sections of SMGs of irradiated Group II sacrificed one month after stopping radiation revealed severe parenchymal atrophy, replaced by fibrous or adipose tissue. The acinar cell nuclei showed significant variation in shape and size (poikilocytosis), condensed chromatin (pyknosis), and occasional abnormal mitosis. The surviving acini were smaller and widely spaced. Group III revealed two types of histological alterations. The first showed parenchymal atrophy, with less densely packed collagen fibers with remnants of degenerated acini and ducts. The second pattern exhibited proliferative activity, characterized by mitotic figures in the acini and ductal system.

Immunohistochemical evaluation

**Figure 2:**
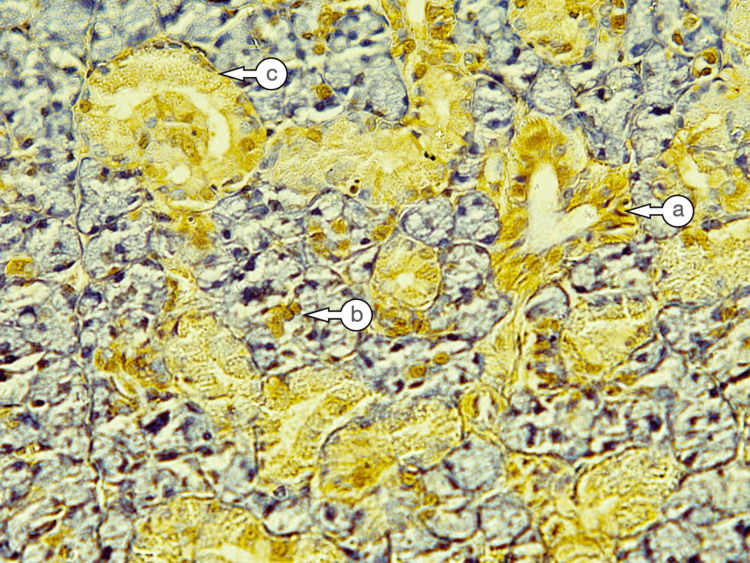
Cytokeratin 17 expression in Group I showed mild diffuse expression in the striated ducts (a), weak expression in the serous acini (b), and mild expression in the granular convoluted tubules (c) (Immuno-peroxidase, magnification X 200).

Control SMG sections exhibited mild expressions in various ductal and serous cells, while the mucous acini showed no staining. Two patterns exhibited CK17 expression: the first was diffused and uniformly distributed across the entire cell cytoplasm, whereas the second showed expression at the lateral and basal parts of the cell, with weaker expression at the portion facing the lumen (Figure 2). In several sections, the main excretory duct displayed mild to moderate expression of the basal cell layer, with weak expression in the superficial layers. The cells of the granular convoluted tubules showed a mild staining reaction with a diffuse pattern.

**Figure 3:**
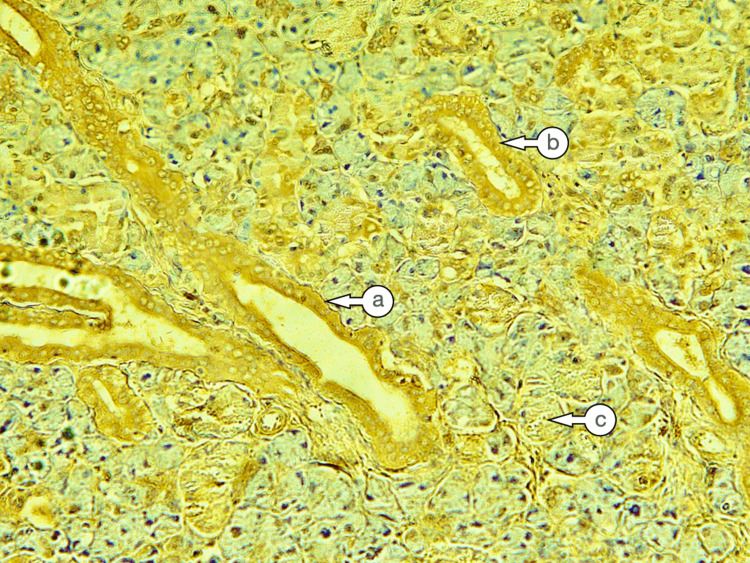
Cytokeratin 17 expressions in Group II showed strong expression in the luminal part and moderate expression at the basal part of striated ducts (a), diffused strong expression in the striated duct (b), and mild expression in the serous acini (c) (Immuno-peroxidase, magnification X 100).

Irradiated Group II revealed CK17 expression varying from mild to strong staining of the intralobular ducts (intercalated and striated) (Figure 3). The staining pattern was either diffuse or concentrated in the luminal cytoplasmic region, with mild staining at the basal portion. Some excretory ducts exhibited strong expression of CK17, either concentrated at the cell apex with moderate staining in the basal portion or diffusely distributed throughout the cell. The serous acinar cells displayed moderate diffuse CK17 expression, while mucous acini showed no staining. Some degenerated ducts and acini showed mild expression.

**Figure 4:**
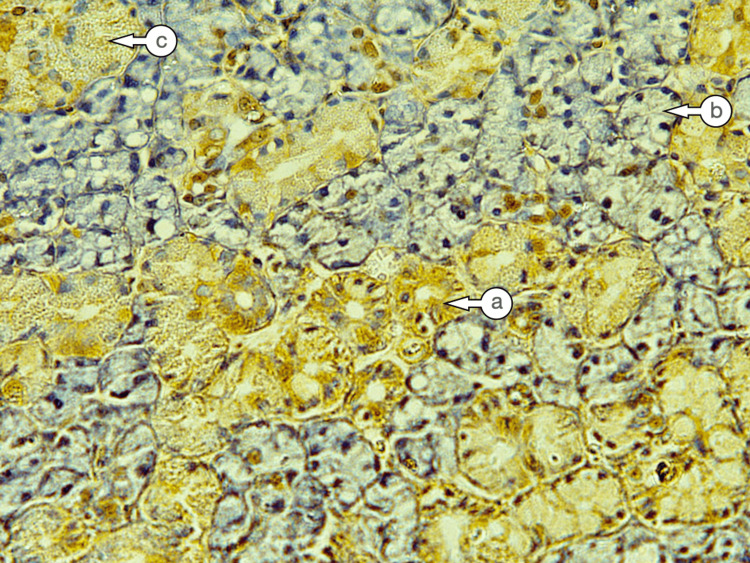
Cytokeratin 17 expression in Group III showed mild to moderate expression in the ducts (a), negative to weak expression in the serous acini (b), and mild expression in the granular convoluted tubules (c) (Immuno-peroxidase, magnification X 200).

The irradiated Group III demonstrated mild to strong expression in the cells of the duct and serous acini. In most specimens, the CK17 staining was diffusely distributed throughout the cytoplasm. In cases with a less distinct pattern, staining was related to the luminal cytoplasmic region, with mild staining observed in the basal portion (Figure 4). Moderate CK17 expression was noted in some excretory ducts and degenerated acini, while the mucous acini showed no staining.

**Figure 5:**
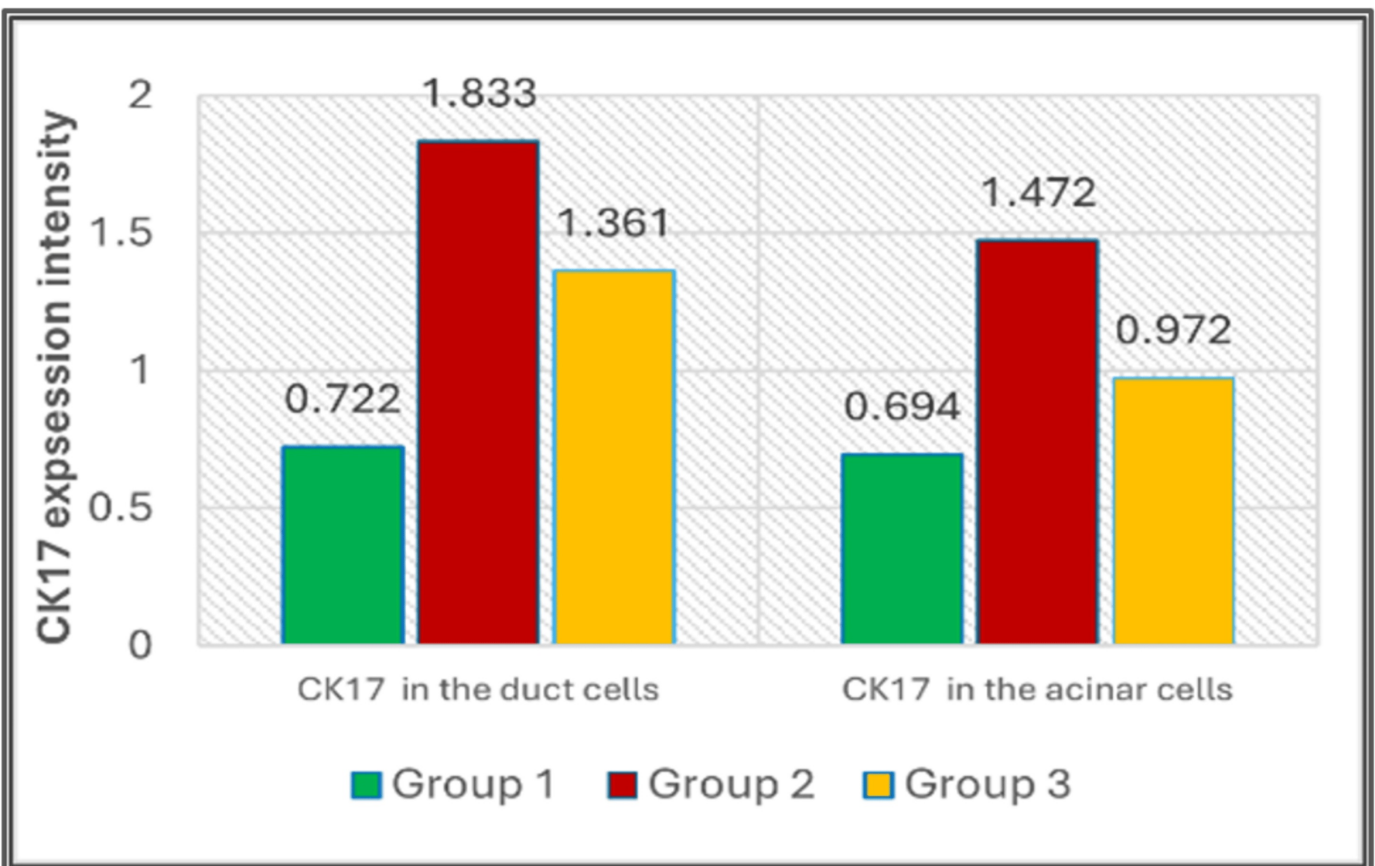
Chart for CK17 expression of both duct and acinar cells among groups CK17: Cytokeratin 17

Statistical summary for all groups, encompassing the mean, median, and standard deviation, is provided in Figure 5 and Table 1. Based on the ANOVA test, analysis of CK17 expression levels in duct cells revealed significant differences (P = 0.00). Additionally, CK17 expression in Group III remained higher than in Group I, with similar significance in the ANOVA test and lower significance in the Tukey HSD test (Tables 2, 3). Cytokeratin expression in Group III was lowered compared to Group II without significance.

**Table 1:** Group statistics of cytokeratin 17 expressions of all variables.

Groups		N	Mean	Std. Deviation	Std. Error Mean
Expression of Cytokeratin 17 in the ductal cells	Group I	9	.7222	.31732	.10577
	Group II	9	1.8333	.62500	.20833
	Group III	9	1.3611	.45262	.15087
	Total	27	1.3056	.65535	.12612
Expression of Cytokeratin 17 in the acinar cells	Group I	9	.6944	.30046	.10015
	Group II	9	1.4722	.49124	.16375
	Group III	9	.9722	.34106	.11369
	Total	27	1.0463	.49535	.09533

**Table 2:** Test of analysis of variance on cytokeratin 17 expression of both duct and acinar cells among groups.

Grouping		Sum of Squares	df	Mean Square	F	Sig.
Expression of Cytokeratin 17 in the ductal cells	Between groups	5.597	2	2.799	12.060	.000
	Within groups	5.569	24	.232		
	Total	11.167	26			
Expression of Cytokeratin 17 in the acinar cells	Between groups	2.796	2	1.398	9.364	.001
	Within groups	3.583	24	.149		
	Total	6.380	26			

**Table 3:** Tukey HSD multiple comparisons on the paired groups. HSD: Honestly significant difference The asterisk (*) in "Mean Difference (I-J)" indicates statistical significance (p ≤ 0.05) between each group and the others.

Tissue of expression	(I) Groups	(J) Groups	Mean Difference (I-J)	Std. Error	Sig.	95% Confidence Interval	
						Lower Bound	Upper Bound
Expression of CK17 in the ductal cells	Group I	Group II	-1.11111*	0.22709	0	-1.6782	-0.544
		Group III	-.63889*	0.22709	0.025	-1.206	-0.0718
	Group II	Group I	1.11111*	0.22709	0	0.544	1.6782
		Group III	0.47222	0.22709	0.115	-0.0949	1.0393
	Group III	Group I	.63889*	0.22709	0.025	0.0718	1.206
		Group II	-0.47222	0.22709	0.115	-1.0393	0.0949
Expression of CK17 in the acinar cells	Group I	Group II	-.77778*	0.18215	0.001	-1.2327	-0.3229
		Group III	-0.27778	0.18215	0.297	-0.7327	0.1771
	Group II	Group I	.77778*	0.18215	0.001	0.3229	1.2327
		Group III	.50000*	0.18215	0.029	0.0451	0.9549
	Group III	Group I	0.27778	0.18215	0.297	-0.1771	0.7327
		Group II	-.50000*	0.18215	0.029	-0.9549	-0.0451

A statistical analysis of CK17 expression levels in acinar cells was conducted using ANOVA, and Tukey tests revealed significant differences (P = 0.001). According to the Tukey test, the increase in CK17 expression in Group III was statistically significant compared with Group II (Tables 2, 3). Group III showed lower CK17 expression than Group II, with a marginal significance, suggesting a reduced cytokeratin intensity.

Tukey HSD multiple comparisons in paired groups showed a significant difference in CK17 expression in the duct system between the control and the irradiated groups (P-value = 0.000 for Group II and P-value = 0.025 for Group III). The discrepancy in P values ​​between the two irradiated groups suggests that although Group III shows less significant results, it is trending toward the expression rate of the control group. Furthermore, additional time may be required for the P-value to become non-significant. In contrast, the cytokeratin expression results in acini showed more advanced progress, with a P-value of 0.297 indicating no significant difference between the control and the third target group. This suggests that regeneration may occur in the serous acini faster than in the duct system.

## Discussion

Salivary gland damage is a well-established consequence of fractionated RT for head and neck cancer, yet pathogenesis remains obscure. Histopathological analysis of the SMG one year after the final radiation dose revealed sustained atrophy and structural damage consistent with Group II findings and underscored the long-term impact of radiation. These findings align with studies reporting persistent salivary gland damage in 65-90% of patients exposed to cumulative radiation doses above 30 Gy [25,26]. The persistent pathological changes observed in our study differ from reports indicating that gland parenchyma heals within two months following radiation [3,17]. Furthermore, Mata et al. (2004) proposed that the survival of smaller ducts and acini within the parenchymal elements could contribute to its regenerative potential [27]. Hassan and Bamaga (2023) noted that smaller acinar cells might act as a defense mechanism, entering a resting state with reduced function to withstand potential damage [28]. Our findings revealed large cells with vacuolated cytoplasm and enlarged nuclei containing distinct mitotic figures, suggesting potential glandular healing or the early stages of abnormal mass formation, as observed by other authors [29,30].

Cytokeratin expression in the control gland exhibited more intense staining in the duct cells than in the serous cells, suggesting that acinar cells are highly differentiated and have fewer intermediate filaments to support intracellular saliva transport and maintain cell architecture [31-33]. Our results' varying CK17 distribution patterns of the control group likely reflect glandular activity, with a diffuse pattern indicating a resting state and reduced expression in the apical region linked to exocytosis. The irradiated groups exhibited markedly stronger staining of CK17 in the duct system and serous acini, likely due to the disruption and aggregation of intermediate filaments within the cytoplasm. Two cytokeratin expression patterns were observed in irradiated glands: a luminal pattern, suggesting early radiation damage, and a diffuse pattern, indicating a more advanced stage, as noted by Hassan and Alqahtani (2024) [25]. While many studies have focused on stem cell research, the nervous system's role, and medical treatments for salivary gland regeneration after radiation, our research examines the gland's healing process after tumor treatment stops, detected by intracellular cytokeratin redistribution [10,34].

Statistical analysis of our study showed that CK17 expression persisted in ductal cells in the irradiated groups compared to the control group. However, a non-significant decrease was observed between the irradiated groups, indicating a slower recovery trajectory. In contrast, the recovery state for acinar cells appears more advanced, with no significant differences in CK17 expression between Group III and Group I. This finding reflects the dynamic nature of acinar cells, which are specialized for secretion and may exhibit faster recovery to restore glandular function. The analysis highlights cellular heterogeneity in tissue recovery, with ductal cells showing initial signs of recovery while acinar cells are at a more advanced stage within the same timeframe. This suggests differing responses to injury and repair timelines, ultimately leading to inevitable healing.

Study limitations

Radiation doses could not be administered for more than six days, as signs of fatigue were observed in the rats on the sixth day, which led to the assumption that they could not tolerate additional doses. Additionally, several mice died on the first day of radiation, which was attributed to the radiation dose. Thus, the dose was reduced from the second day for the remaining radiation sessions. One of the key limitations of the experiment was the inability to extend the waiting period beyond a year, as the rats were approaching the end of their lifespan.

## Conclusions

In conclusion, CK17 immunostaining showed distinct expression patterns in control and irradiated SMGs, indicating the relationship between cytokeratin distribution and gland activity. In irradiated glands, CK17 staining was significantly stronger in the duct and serous cells, impairing saliva production, transport, and the exocytosis pathway. The statistical analysis of CK17 expression in irradiated groups shows differing recovery times between ductal and acinar cells, in which acinar cells exhibit more advanced recovery than ductal cells. Therefore, it is recommended that oncologists allow the salivary glands to heal naturally and avoid excessive use of diuretics after radiation unless necessary. Further research on animals with longer lifespans is needed to understand the resolution of pathological changes in the SMGs.
